# Acute Exacerbation of Idiopathic Pulmonary Fibrosis With Concurrent Cardiac Amyloidosis: A Technetium Pyrophosphate Study

**DOI:** 10.7759/cureus.56358

**Published:** 2024-03-18

**Authors:** Toyoshi Yanagihara, Hikaru Hatashima, Hiroaki Ogata, Yuki Moriuchi, Akiko Ishimatsu, Junji Otsuka, Kazuhito Taguchi, Atushi Moriwaki, Makoto Yoshida

**Affiliations:** 1 Department of Respiratory Medicine, National Hospital Organization (NHO) Fukuoka National Hospital, Fukuoka, JPN; 2 Department of Cardiology, National Hospital Organization (NHO) Fukuoka National Hospital, Fukuoka, JPN

**Keywords:** technetium pyrophosphate scintigraphy, hfpef, cardiac amyloidosis, acute exacerbation, idiopathic pulmonary fibrosis

## Abstract

Amyloidosis presents a diagnostic challenge, particularly when concomitant with severe conditions like acute exacerbations of idiopathic pulmonary fibrosis (IPF). In this report, we detail the case of a 73-year-old patient with acute exacerbation of IPF and simultaneous emergence of cardiac amyloidosis. The patient's clinical journey began with persistent exertional dyspnea, progressing to hypoxemia on admission. Chest CT scans showed extensive ground-glass opacities, consolidations, and pre-existing honeycombing-like cysts and reticular shadows, accompanied by a right-sided pleural effusion. The therapeutic strategy for acute exacerbation of IPF encompassed methylprednisolone pulse therapy, tacrolimus, and nintedanib, augmented with intravenous immunoglobulin and recombinant thrombomodulin. Concurrently, heart failure with preserved ejection fraction was managed with a pharmacological trio: empagliflozin, diuretics, and eplerenone. A hypertrophied heart and low limb voltage prompted an investigation for cardiac amyloidosis, which ^99m^Technetium pyrophosphate (^99m^Tc-PYP) scintigraphy confirmed, yielding a probable diagnosis. Following steroid tapering, the patient was discharged home. This case prompted an investigation into the potential role of amyloidosis in pulmonary pathology. Our retrospective review of 10 patients, including four with cardiac amyloidosis, who underwent ^99m^Tc-PYP scintigraphy, revealed a nonsignificant yet notable trend of increased pulmonary accumulation in cardiac amyloidosis cases (median (interquartile range): 5.4×10^4 ^(5.3-13.1×10^4^) vs. 3.6×10^4 ^(2.4-5.1×10^4^), p=0.0667). Notably, the pulmonary counts in this patient exceeded the negative cohort's mean values, hinting at a possible contribution of amyloid deposition to pulmonary pathology. This study, pioneering in evaluating lung field accumulation of ^99m^Tc-PYP in cardiac amyloidosis, may provide novel insights into the influence of amyloidosis on pulmonary conditions.

## Introduction

Idiopathic pulmonary fibrosis (IPF) is a chronic, progressive lung disease characterized by fibrotic changes within the lung parenchyma. The prognosis post-diagnosis typically ranges between three and five years [1,2]. The prognosis is notably worsened, with a median of three to four months by the occurrence of acute exacerbations (AEx) [3]. These exacerbations are correlated with a significant decline in lung function and an increase in mortality, highlighting the critical need for effective therapeutic strategies.

Amyloidosis is a group of chronic degenerative diseases marked by the deposition of misfolded proteins, which can affect various organs such as the heart, kidneys, gastrointestinal tract, respiratory system, and nervous system [4]. Deposits in the heart that disrupt cardiac function and rhythm are known as cardiac amyloidosis [5]. Cardiac amyloidosis is particularly challenging to diagnose due to the non-specific nature of initial investigative modalities such as echocardiography and electrocardiography, which often yield findings indistinguishable from other cardiac disorders. However, ^99m^Technetium pyrophosphate (^99m^Tc-PYP) scintigraphy has proven to be a superior diagnostic tool for detecting transthyretin (ATTR) amyloidosis [5]. Imaging performed one or three hours post-injection of ^99m^Tc-PYP that demonstrates cardiac uptake suggests a strong suspicion of ATTR amyloidosis, which may then be confirmed through endomyocardial biopsy. Consequently, ^99m^Tc-PYP scintigraphy is an essential non-invasive examination within the diagnostic pathway for ATTR cardiac amyloidosis. Although pulmonary involvement in amyloidosis is less frequent, it presents significant diagnostic challenges due to the non-specific nature of its radiological features and the scarcity of antemortem diagnosis [6,7].

Here, we present a patient treated for an AEx-IPF, during which cardiac amyloidosis was concurrently diagnosed. The clinical AEx-IPF was made in the absence of histopathological confirmation. Given the intriguing possibility of pulmonary amyloidosis contributing to the disease process, we conducted a retrospective analysis of 10 cases at our institution in which ^99m^Tc-PYP scintigraphy was employed for suspected cardiac amyloidosis. This analysis aimed to evaluate the extent of 99mTc-PYP uptake in the lung fields of patients with cardiac amyloidosis.

## Materials and methods

Study participants

This study was conducted on a selected cohort of individuals aged 20 years or older who were subjected to ^99m^Tc-PYP scintigraphy at our institution, National Hospital Organization (NHO) Fukuoka National Hospital, in Fukuoka, Japan, prompted by a clinical suspicion of cardiac amyloidosis. The timeframe for this retrospective analysis spanned from May 1, 2020, to November 30, 2023. A review of existing patient records was undertaken to collate pertinent clinical data. Ethical clearance for this investigation was granted by the Institutional Review Board of the National Hospital Organization (NHO) Fukuoka National Hospital under the approval number F5-31. The research adhered to the Helsinki Declaration guidelines. Owing to the retrospective design of the study, the requirement for informed consent was waived. However, written informed consent was obtained from the patient for case presentation. Patient data was anonymized to maintain the confidentiality and privacy of the participants.

^99m^Tc-PYP scintigraphy

﻿^99m^Tc-PYP was administered intravenously in 10-20 seconds, followed by a 10-mL saline flush to ensure complete trace delivery. The administered isotope dose was 476 MBq (12.86 mCi). Imaging acquisition utilized a conventional gamma camera, with scans executed one hour after injection. (Imaging was also acquired three hours after injection for the later phase.) The semiquantitative evaluation uses a heart-to-rib comparison scale: grade 0 indicates no cardiac uptake with standard rib uptake; grade 1 reflects cardiac uptake lower than rib uptake; grade 2 denotes cardiac uptake on par with rib uptake; and grade 3 represents cardiac uptake surpassing rib uptake, accompanied by diminished or absent rib uptake. Quantitative assessment is conducted by comparing average counts obtained from a region of interest over the cardiac area to those from a region of equivalent size and intensity over the contralateral thoracic area. The ^99m^Tc-PYP scan is diagnostic of ATTR cardiac amyloidosis if there is grade 2 to 3 cardiac uptake or a heart/contralateral (H/CL) ratio >1.5 [8]. Quantification of pulmonary ^99m^Tc-PYP uptake was determined by averaging counts from the right upper, right lower, and left upper lung fields.

Statistical analysis

The Mann-Whitney test was utilized to perform a comparative analysis between the two groups using GraphPad Prism 9 software. A p-value less than 0.05 was considered to indicate statistical significance.

## Results

Case presentation

A 73-year-old male with a seven-year history of incidental radiological findings indicative of interstitial lung disease (ILD) was admitted with a month-long exacerbation of exertional dyspnea, culminating in significant respiratory distress (modified Medical Research Council (mMRC) grade 3) the day before the presentation. The patient's past medical history was notable only for smoking (20 cigarettes per day for 30 years until cessation at age 50), with no reported comorbidities, medication use, or occupational dust exposure.

Initial examination revealed a height of 165 cm, a weight of 70 kg, and a body mass index (BMI) of 25.7 kg/m². Vital signs showed a heart rate of 74 bpm, a blood pressure of 137/84 mmHg, a temperature of 37.1°C, a respiratory rate of 24 /min, and an oxygen saturation of 89% on room air, which improved to 95% at rest. Physical examination revealed late inspiratory crackles in the bilateral dorsolateral lower lung fields with no cardiac murmurs. Jugular venous distention, edema, joint pain, and rash were absent.

**Figure 1:**
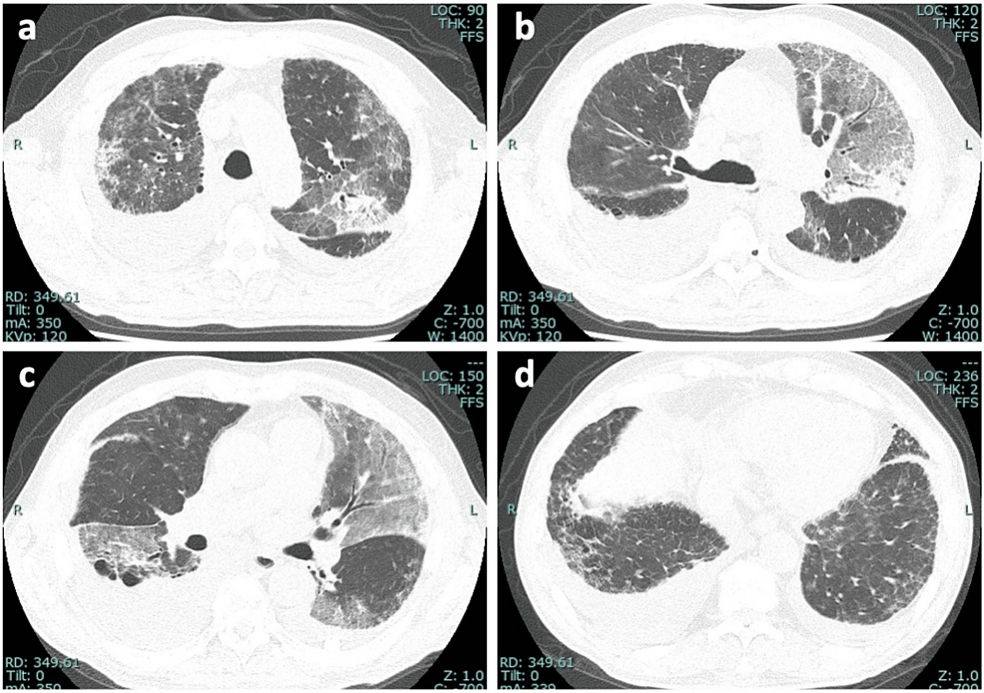
Chest CT images of the patient on admission. (a-d) A high-resolution chest CT scan shows diffuse ground-glass opacities and some consolidations overlying known honeycombing-like cysts and reticular shadows, with concurrent right-sided pleural effusion.

**Figure 2:**
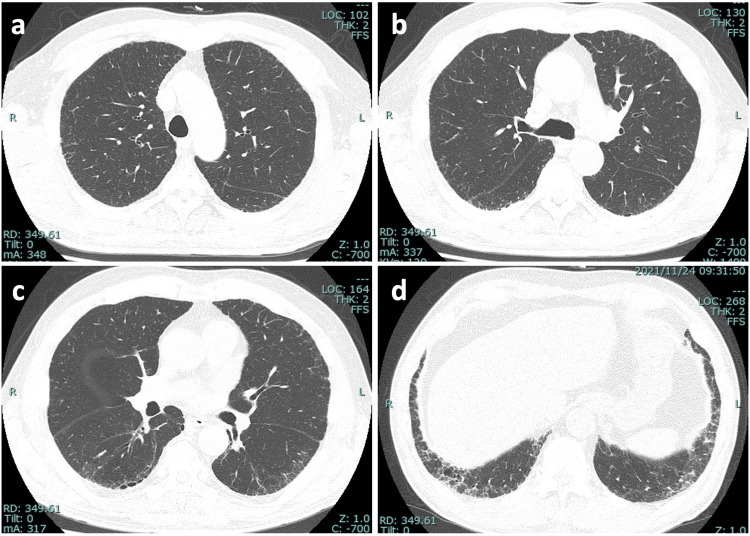
Chest CT images of the patient two years before admission. (a-d) A high-resolution chest CT scan taken two years prior to the patient's admission reveals a mild, subpleural, and basally predominant reticular pattern, accompanied by traction bronchiectasis and bronchiolectasis across both lungs, indicative of a probable UIP pattern. UIP: usual interstitial pneumonia

**Figure 3:**
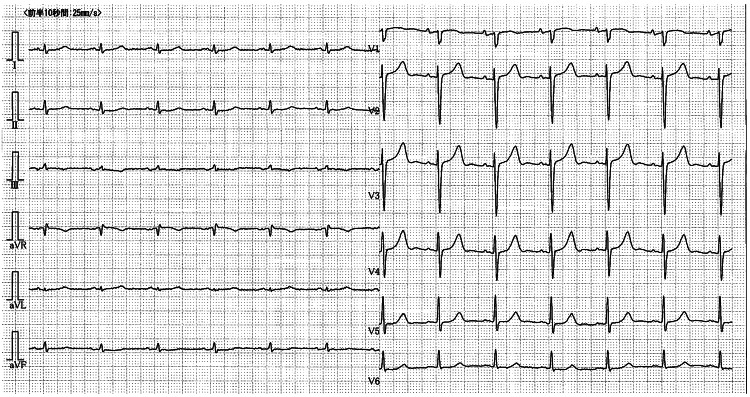
The electrocardiogram of the patient.

Laboratory tests showed elevated levels of C-reactive protein (CRP) (11.81 mg/dL), lactate dehydrogenase (LDH) (293 U/L), D-dimer (11.2 µg/mL), N-terminal pro-brain natriuretic peptide (NT-proBNP) (3,598 pg/mL), and Krebs von den Lungen 6 (KL-6) (716 U/mL). A chest CT scan demonstrated diffuse ground-glass opacities and some consolidations overlying known honeycombing-like cysts and reticular shadows, with concurrent right-sided pleural effusion (Figure 1), which largely differed from CT findings two years earlier (Figure 2). Nasopharyngeal FilmArray was negative. The electrocardiogram revealed normal sinus rhythm with low voltage in limb leads (Figure 3). Echocardiography revealed a left ventricular ejection fraction (LVEF) of 61%, but an elevated E/A ratio of 3.90 and E/E’ of 13.4 suggested left ventricular diastolic dysfunction. Furthermore, diffuse left ventricular hypertrophy was noted with LVDd/s at 46/31 mm and LAD/AoD at 46/32 mm.

**Figure 4:**
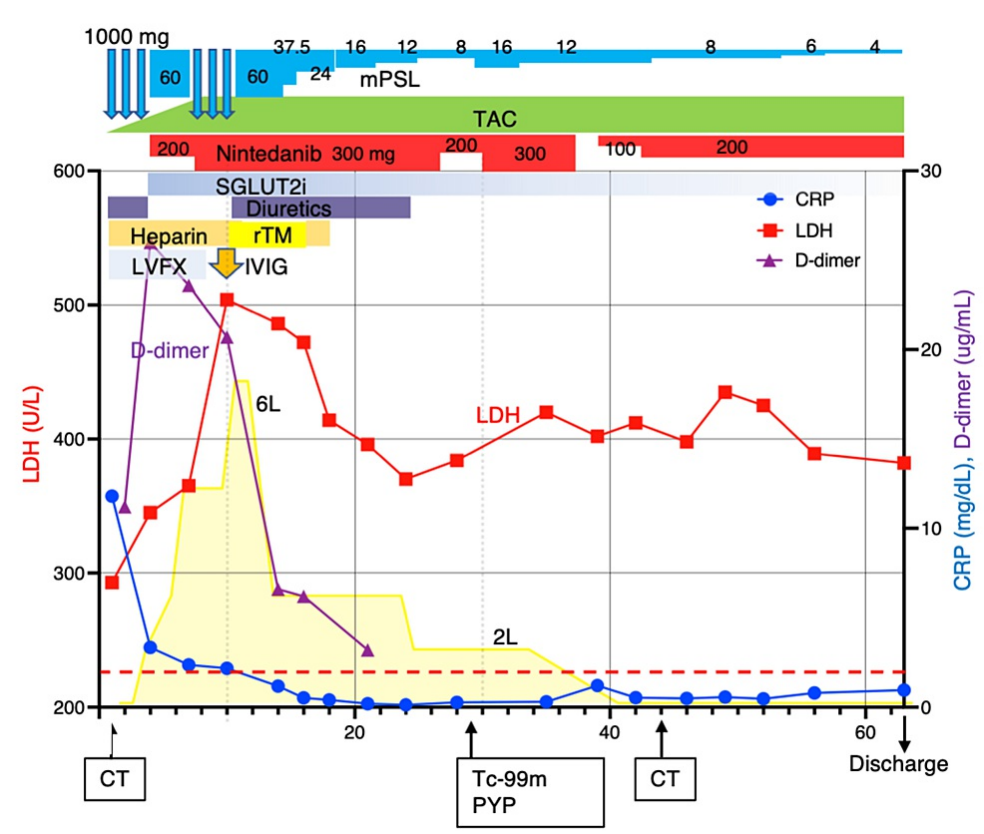
The clinical course of the patient. This timeline delineates the patient's clinical progression, therapeutic interventions, and response to treatment over the course of hospitalization. Key biomarkers and oxygen supplementation and adjustments in medication dosages are chronicled in relation to the corresponding hospital day. mPSL: methylprednisolone; LVFX: levofloxacin; TAC: tacrolimus; SGLUT2i: sodium-glucose transporter 2 inhibitor; IVIG: intravenous immunoglobulin; Tc-99m PYP: ^99m^Technetium pyrophosphate

The patient was managed for AEx of ILD and heart failure with preserved ejection fraction (HFpEF), receiving a comprehensive treatment regimen including high-dose methylprednisolone (mPSL), tacrolimus, levofloxacin, nintedanib, anticoagulants, diuretics, and empagliflozin (Figure 4). Despite initial treatment, respiratory failure progressed, with persistent elevations in serum LDH levels, suggesting AEx of ILD progressed. On day 7, pulse-dose mPSL was again administered. On day 10, the LDH level reached 504 U/L, with a significant increase of D-dimer at 20.7 µg/mL and fibrin degradation product (FDP) at 34.8 µg/mL without evidence of deep venous thrombosis. Additional administration of recombinant thrombomodulin (rTM) 25,600 units/day and 10 g of intravenous immunoglobulin (IVIG) along with diuretics and eplerenone led to clinical improvement. A clinical diagnosis of IPF was established based on medical history, imaging, and serological findings measured on admission (absence of any autoantibodies) (Table 1).

**Table 1:** Serological examination of the patient. RF: rheumatoid factor; ARS: aminoacyl-tRNA synthetase; PR3-ANCA: proteinase-3-antineutrophil cytoplasmic antibody; MPO-ANCA: myeloperoxidase-antineutrophil cytoplasmic antibody

Test	Value	Unit	Reference
Anti-nuclear antibody	<40	Titers	<40
RF	4	IU/mL	<15
Anti-ARS antibody	5.4	Index	<24
Anti-SS-A antibody	<1.0	U/mL	<10
Anti-Scl-70 antibody	<1.0	U/mL	<10
PR3-ANCA	<1.0	IU/mL	<2.0
MPO-ANCA	<1.0	IU/mL	<3.5
Bird-specific antibody	Negative	mgA/L	
Anti-*Trichosporon asahii* antibody	Negative	CAI	<0.15

**Figure 5:**
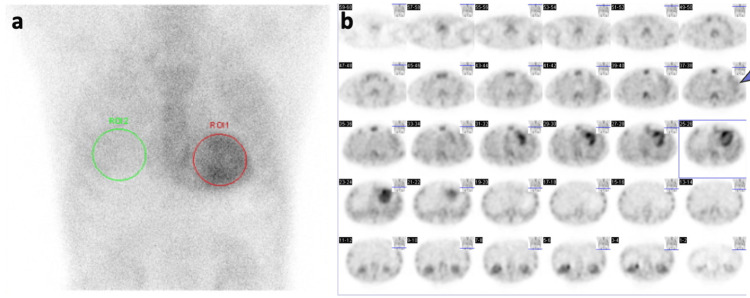
99mTechnetium pyrophosphate scintigraphy of the patient. (a) Anteroposterior scintigraphy view displaying cardiac uptake. The red circle delineates the myocardial region, and the green circle demarcates the contralateral reference area. (b) Sequential axial slices of ^99m^Technetium pyrophosphate scintigraphy performed one hour post-injection. An arrowhead highlights the notable radiotracer uptake in the patient's left upper pulmonary lobe.

A hypertrophied heart and low limb voltage warranted an investigation for cardiac amyloidosis. On day 29, ^99m^Tc-PYP scintigraphy was conducted. ^99m^Tc-PYP was accumulated in the left ventricle (visual grade 3, H/CL ratio at one hour: 2.12) (Figure 5), confirming a probable diagnosis of cardiac amyloidosis.

**Figure 6:**
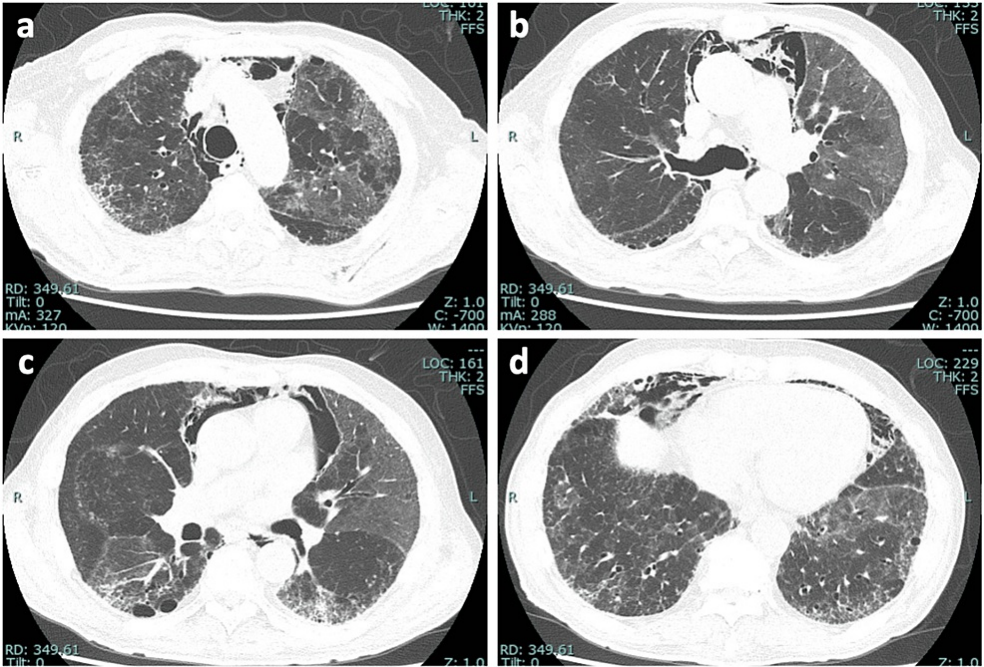
Following chest CT images of the patient on day 44. (a-d) The sequential panels from a high-resolution chest CT demonstrate marked improvements in ground-glass opacities and consolidations. Persistent honeycombing-like cysts and reticular patterns are visible, predominantly within the basal segments of the lungs. Mediastinal emphysema is also evident.

The hospital course was complicated by mediastinal/subcutaneous emphysema and nintedanib-related diarrhea (grade 2), managed by temporary discontinuation and dose adjustment of nintedanib. Trough levels of tacrolimus intended to control within 5-10 ng/mL. On day 44, subsequent chest CT images showed improvement in ground-glass opacities and infiltrations (Figure 6). mPSL was further tapered, and the patient was discharged home on day 63. Subsequent referral to a tertiary center confirmed wild-type ATTR cardiac amyloidosis through endomyocardial biopsy and genetic analysis.

A trend of higher technetium pyrophosphate accumulation in the lung field in patients with cardiac amyloidosis

**Figure 7:**
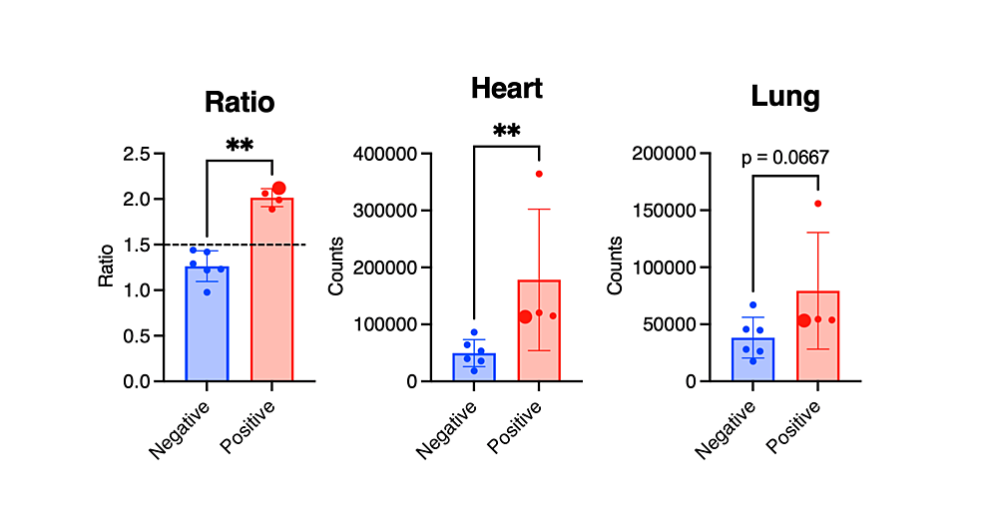
Comparative analysis of 99mTechnetium pyrophosphate scintigraphy parameters in cardiac amyloidosis. This figure delineates the H/CL ratio and the ^99m^Technetium pyrophosphate counts within the cardiac and pulmonary fields among patients grouped by the presence or absence of cardiac amyloidosis. The enlarged dots signify the subject detailed in the case presentation. A double asterisk (**) indicates statistical significance with a p-value less than 0.01. H/CL: heart-to-contralateral

^99m^Tc-PYP scintigraphy was conducted for 10 cases from May 1, 2020, to November 30, 2023, at our institution. Of these, four cases were positive, while six cases were negative (Table 2). The mean age, BMI, NT-proBNP, and LVEF for the positive group and the negative group were not different between groups (Table 3). The H/CL ratio was 2.03 (1.92-2.11) in the positive group and 1.26 (1.16-1.43) in the negative group, as the cut-off of the H/CL ratio was set to 1.5 (Figure 6). Heart counts were much higher in the positive group compared to the negative group (11.8×104 (11.4-30.3×104) vs. 4.6×104 (3.1-7.0×104), p=0.0095) (Figure 7). Interestingly, there was a tendency of increased lung counts in the positive group compared to the negative group (5.4×104 (5.3-13.1×104) vs. 3.6×104 (2.4-5.1×104), p=0.067) (Figure 7). Notably, the pulmonary counts in this patient exceeded the negative cohort's mean values, hinting at a possible contribution of amyloid deposition to pulmonary pathology. Specifically, the count in the left upper lobe of the lung seemed to be increased (arrowheads in Figure 5), where extensive ground-glass opacities remained on the following chest CT images.

**Table 2:** Baseline characteristics of the patients in the cohort study. This table summarizes the baseline demographic and clinical characteristics of patients enrolled in the cohort study. It presents individual data on age, sex, height, weight, BMI, NT-proBNP levels, LVEF, the H/CL ratios of ^99m^Tc-PYP uptake, and actual counts in heart and lung regions.  Patients identified as P1-P4 are those with a positive diagnosis of cardiac amyloidosis, while N1-N6 represent the non-amyloidosis cases. BMI: body mass index; NT-proBNP: N-terminal pro-brain natriuretic peptide; LVEF: left ventricular ejection fraction; H/CL: heart-to-contralateral; 99mTc-PYP: ^99m^Technetium pyrophosphate

Case	Age	Sex	Height (cm)	Weight (kg)	BMI (kg/m^2^)	NT-proBNP (pg/mL)	LVEF (%)	^99m^Tc-PYP		
								H/CL ratio	Heart count	Lung count
P1	73	M	165	70	25.71	3598	67	2.12	113181	53201
P2	86	M	173	43.5	14.53	7850	54	1.89	120544	54380
P3	86	M	168	51	18.06	2870	35	1.99	364337	155947
P4	84	M	155	52	21.64	4000	38	2.06	115007	53768
N1	88	M	167	61	21.87	1470	53	1.44	39783	28051
N2	76	F	146	54	25.33	5950	71	1.23	86199	67061
N3	77	M	152	64	27.70	901	81	0.98	18622	17525
N4	77	M	163	62	23.33	2234	66	1.22	53320	44773
N5	67	M	173	68	22.72	1119	53	1.29	35771	26381
N6	69	M	160	69	26.95	807	63	1.42	64322	45601

**Table 3:** Summary of the characteristics of the study. Data are presented as mean±standard deviation. BMI: body mass index; LVEF: left ventricular ejection fraction; H/CL: heart-to-contralateral

	Negative (n=6)	Positive (n=4)	P-value
Age	75.6±7.4	82.0±6.7	0.319
BMI	24.7±2.4	20.0±4.8	0.1143
NT-proBNP (pg/mL)	2080±1965	4580±2230	0.067
LVEF(%)	64.5±10.8	48.5±14.9	0.2286
H/CL ratio	1.26±0.17	2.02±0.10	0.0095
Heart count	49670±23737	178267±124086	0.0095
Lung count	38232±17895	79324±51084	0.067

## Discussion

In the case presentation, we have shown that AEx-IPF coexists with cardiac amyloidosis. Currently, there are insufficient established evidence-based treatments for AEx-IPF. International guidelines offer a weak recommendation for the use of systemic steroids during IPF-AEx [9]. However, the dosage and administration of systemic steroids for AEx-IPF vary by institution and region, with evidence for appropriate dosing still markedly insufficient. A recent retrospective observational study analyzed 238 AEx-IPF patients treated with corticosteroids at a tertiary referral hospital [10]. Patients were categorized into pulse or non-pulse corticosteroid regimen groups based on administering a pulse dose (≥250 mg/day of mPSL or equivalent) within the first seven days of hospitalization. Of the 238 patients, 59 received a pulse corticosteroid dose, while 179 were given a conventional non-pulse dose. After adjusting for confounders related to baseline clinical and radiographic severity, the pulse regimen did not significantly reduce mortality risk at three-month (aHR 0.84, 95% CI 0.45-1.38) or 12-month (aHR 0.96, 95% CI 0.60-1.25) follow-ups compared to the conventional non-pulse regimen. A Japanese nationwide study also compared high-dose (500-1,000 mg/day of mPSL within the first four days of admission for three days) versus low-dose (100-200 mg/day within the same timeframe for at least five days) regimens for AEx of idiopathic interstitial pneumonia, concluding no significant difference in in-hospital mortality rates [11]. In our case, we administered mPSL at 1,000 mg twice for three days; however, these studies suggest that such high dosing may not have been necessary.

We administered nintedanib very early (on day 4) for the case. Recently, a Japanese nationwide study showed that nintedanib initiation within 14 days post-admission during AEx of fibrosing ILDs was significantly associated with a lower risk of in-hospital death and shorter length of hospitalization in patients with fibrosing ILDs [12]. There are also case series where patients with AEx-ILDs were successfully managed with the early administration of nintedanib within 14 days post-admission [13]. Based on the literature, it seems to be better to administer nintedanib at the acute phase of AEx-ILDs.

Regarding the administration of rTM for AEx-IPF, a randomized, double-masked, placebo-controlled trial was reported [14]. rTM did not improve the 90-day survival proportion. The present results suggest that the use of rTM for treating AEx-IPF is not recommended [14]. On the contrary, higher FDP (>10 µg/dL) was reported to be the poor outcome of AEx of idiopathic interstitial pneumonias, and rTM improved the survival of such patients, though retrospective nature [15]. In this case, the FDP level reached 34.8 µg/mL regardless of treatment with heparin calcium on day 10, which satisfied the criteria of the Japanese Associate for Acute Medicine disseminated intravascular coagulation (JAAM-DIC) [16]. After the initiation of rTM, the respiratory condition, LDH levels, and FDP levels were all improved. Based on the results of the randomized clinical trial [14], we should not use rTM for AEx-IPF in general. However, rTM may be the one option for cases with AEx-ILDs that satisfy the criteria of the JAAM-DIC.

Regarding the coexistence of AEx-IPF and HFpEF in the representative case, we hypothesize that the exacerbation-induced pulmonary overload may have unmasked latent diastolic dysfunction exacerbated by underlying cardiac amyloidosis. The rarity of case reports connecting AEx-IPF with ATTR cardiac amyloidosis may be attributed to the recent advent of ^99m^Tc-PYP scintigraphy as a diagnostic tool for ATTR heart amyloidosis. This advancement suggests the possibility of undiagnosed concurrent IPF and ATTR cardiac amyloidosis in previous cases. Regarding parenchymal changes, we acknowledge that these alterations could result from amyloid deposition or inflammatory processes associated with AEx-IPF. It is noteworthy that ^99m^Tc-PYP has been documented to accumulate at sites of inflammation, as evidenced in studies where 99mTc-PYP accumulation was observed in the muscles of patients with idiopathic inflammatory myopathies [17]. Although the ^99m^Tc-PYP scintigraphy in our case was performed on day 29 post-admission, when inflammation was presumably reduced, residual inflammatory activity in the lungs cannot be ruled out.

Our analysis revealed a trend of elevated ^99m^Tc-PYP counts in the pulmonary fields among patients with cardiac amyloidosis compared to those without. Research on systemic soft tissue uptake of phosphonate-based radiotracers via nuclear scintigraphy has predominantly focused on ^99m^Tc-hydroxymethylene diphosphonate (HMDP), a tracer commonly utilized throughout Europe for whole-body scans to detect ATTR cardiac amyloidosis. Malka et al. observed extracardiac ^99m^Tc-HMDP uptake in 46 out of 182 patients with ATTR cardiac amyloidosis, with the majority of uptake found in the pleuropulmonary parenchyma (33/182, 18%), followed by the digestive tract and subcutaneous fat [18]. The Methodological Amyloidosis Diagnostic Index (MADI) developed by Malka et al. correlates extracardiac uptake with poorer patient outcomes, particularly when comparing higher MADI scores indicative of additional extracardiac uptake to those with cardiac uptake alone [18]. These findings suggest a possible correlation with increased ^99m^Tc-PYP uptake in the lung fields despite the use of a different tracer.

As for ^99m^Tc-PYP, Takahashi et al. documented a case of acute decompensated heart failure where ^99m^Tc-PYP concentrated not only in the myocardium but also in extracardiac areas such as the thoracic and abdominal walls, confirmed by a positive fine-needle aspiration biopsy for ATTR amyloidosis [19]. While another study noted that extracardiac uptake of ^99m^Tc-PYP in heart failure was infrequent and seldom necessitated further investigation, it lacked specific data on cardiac amyloidosis, precluding any comparative analysis of uptake levels between patients with and without cardiac amyloidosis [20]. Given this gap, our research provides valuable insights into the patterns of ^99m^Tc-PYP accumulation in the lungs and its implications for extracardiac tissue involvement in cardiac amyloidosis.

A primary limitation of this study is the absence of histopathological confirmation. The diagnosis of cardiac amyloidosis was based primarily on ^99m^Tc-PYP scintigraphy results, rendering it a probable rather than a definitive diagnosis. Similarly, the diagnosis of IPF relied on clinical and radiological evaluations. Histological analysis was not conducted due to the acute exacerbation phase of the disease, where the risks associated with biopsy procedures were deemed considerable. This constraint highlights the need for a cautious interpretation of the findings and underscores the potential value of integrating histological data in future studies.

## Conclusions

In the case presentation, we elucidate the complexities in diagnosing and managing the co-occurrence of AEx-IPF with cardiac amyloidosis. The presented case adds depth to the current discourse on AEx-IPF management strategies, particularly in the context of steroid dosing, nintedanib administration timelines, and the debated efficacy of rTM. A key observation from our research is the pattern of increased ^99m^Tc-PYP uptake in the lung areas of patients with cardiac amyloidosis, enriching our insight into the systemic absorption patterns of phosphonate-based radiotracers. While these findings underscore a potential diagnostic and prognostic value, they underscore the need for further, more detailed investigations to validate these preliminary observations and address any existing biases. Thus, this study acts as an exploratory step towards a deeper, more nuanced understanding of these complex conditions, advocating for future research aimed at elaborating on these initial findings.
